# Taylor's law and body size in exploited marine ecosystems

**DOI:** 10.1002/ece3.418

**Published:** 2012-11-15

**Authors:** Joel E Cohen, Michael J Plank, Richard Law

**Affiliations:** 1 Laboratory of Populations, Rockefeller & Columbia UniversitiesNew York, New York; 2 Department of Mathematics and Statistics, University of CanterburyChristchurch, New Zealand; 3 Department of Biology, University of YorkYork, UK

**Keywords:** Balanced harvesting, density-mass allometry, fishing, power law, size spectrum, size-at-entry, variance-mass allometry

## Abstract

Taylor's law (TL), which states that variance in population density is related to mean density via a power law, and density-mass allometry, which states that mean density is related to body mass via a power law, are two of the most widely observed patterns in ecology. Combining these two laws predicts that the variance in density is related to body mass via a power law (variance-mass allometry). Marine size spectra are known to exhibit density-mass allometry, but variance-mass allometry has not been investigated. We show that variance and body mass in unexploited size spectrum models are related by a power law, and that this leads to TL with an exponent slightly <2. These simulated relationships are disrupted less by balanced harvesting, in which fishing effort is spread across a wide range of body sizes, than by size-at-entry fishing, in which only fish above a certain size may legally be caught.

## Introduction

Two widely observed empirical patterns in ecology are Taylor's law (TL; Taylor 1961; Eisler et al. 2008) and density-mass allometry (Blackburn and Gaston 1999; Jennings et al. 2007; Belgrano and Reiss 2011). TL asserts that, in an ensemble of populations, the variance of the population density is a power-law function of the mean density of those populations. Density-mass allometry asserts that population density is a power-law function of mean body mass, and can refer to single or mixed species as well as to individuals regardless of species. Composing TL with density-mass allometry predicts that the variance of population density should be a power-law function of mean body size and that the parameters of that power law should be predictable from the parameters of TL and of density-mass allometry (Marquet et al. 2005 and, independently, Cohen et al. 2012). Cohen et al. (2012) confirmed this relationship using detailed forestry data.

Taylor's law has been confirmed for hundreds of species or groups of related species in field observations and laboratory experiments (Reed and Hobbs 2004; Benton and Beckerman 2005; Marquet et al. 2005; Eisler et al. 2008; Ramsayer et al. 2011; Kaltz et al. 2012), and numerous models have been proposed to explain TL under various assumptions (e.g. Anderson et al. 1982; Ballantyne IV 2005; Engen et al. 2008). However, there is no consensus about why TL is so widely observed, how its estimated parameters should be interpreted in terms of underlying population dynamics, and when it might fail to be valid.

Density-mass allometry is seen in at least two different contexts. First, the allometry is widely observed across taxa, when each taxon is described by an average body size and a population density, although the exponent of the power law appears to differ for different groups of organisms (Damuth 1981, 1987; Lawton 1989; Marquet et al. 1990; Silva and Downing 1995; Dunham and Vinyard 1997; Enquist et al. 1998; Hendriks 1999; Schmid et al. 2000; Morand and Poulin 2002; Niklas et al. 2003; Makarieva et al. 2005; Reuman et al. 2008, 2009). This form of density-mass allometry is sometimes referred to as Damuth's law. Second, density-mass allometry is often observed using the densities of individuals grouped by body mass irrespective of taxon. In plant ecology, this form of density-mass allometry is called the self-thinning law (Adler 1996); in marine ecosystems, it is called a size spectrum (Sheldon and Parsons 1967), and this is the form of density-mass allometry used in this article.

Although the allometry of density with taxon average body mass (Damuth's law) has been controversial (Marquet et al. 1995, 2005), the allometry of density with individual body mass in marine ecosystems is clearly supported by empirical data showing that the total biomass in logarithmic bins of body mass is approximately the same across a wide range of body sizes (Sheldon et al. 1972, 1977; Boudreau and Dickie 1992; Kerr and Dickie 2001). Equivalently, the density of organisms of a given mass (per unit volume per unit body mass) is a power-law function of body mass with an exponent close to −2 (Sheldon and Parsons 1967; Platt and Denman 1978; San Martin et al. 2006).

Dynamic models of size spectra in marine ecosystems are based on a size-specific account of predation and growth as organisms eat one another, typically using the McKendrick–von Foerster partial differential equation (Benoît and Rochet 2004; Andersen and Beyer 2006; Blanchard et al. 2009; Law et al. 2009; Hartvig et al. 2011; Zhang et al. 2012). Such models are deterministic and can display at least two modes of behavior, depending on parameter values. In the stable mode, the biomass in any bin of body size converges to a fixed limit in time. In the oscillatory mode, the biomass in any bin of body size converges to a periodic cycle. Other dynamics, such as divergence or chaos, may be possible and have not yet been investigated in detail.

Because the oscillatory mode of dynamic size spectrum models predicts temporally varying population densities, it is natural to ask, for each body-size bin, (a) how the variance of population density is related to the mean of population density (does the model obey TL?), (b) how the mean population density is related to body size (does the model obey density-mass allometry?), and (c) how the variance of population density is related to body size (does the model obey variance-mass allometry?). Question (b) has been investigated in depth (Sheldon et al. 1972, 1977; Boudreau and Dickie 1992; Andersen and Beyer 2006), but neither question (a) nor question (c) has. Positive answers to these questions would provide novel interpretations of TL and variance-mass allometry in terms of the dynamic processes in size spectrum models. Negative answers would challenge the application of TL to dynamic size spectrum models.

The relationships among variability, density, and body size in marine ecosystems are important from a practical perspective because fish stocks appear to show increased variation under pressure of fishing (Hsieh et al. 2006; Andersen and Pedersen 2010; Rochet and Benoît 2012). However, the natural scaling of variability with density and body size in these ecosystems is largely unknown. Moreover, the traditional method of managing fisheries, protecting young, small fish and harvesting old, large ones is coming under increasing scrutiny. One suggestion is that moving away from highly selective fishing, and instead spreading the fishing effort widely over species and sizes, would ensure a sustainable fishery while reducing waste and conserving biodiversity. This approach to fishing is referred to as balanced harvesting (Zhou et al. 2010; Garcia et al. 2012). Theoretical results from a size spectrum model suggest that this approach has the potential to reduce the disruption to the natural size structure of the system (Law et al. 2012), but the effects of harvest patterns on the scaling of variability with body size and density remain unknown.

Here, we examined the extent to which TL and variance-mass allometry apply to size spectrum models. We did this first using a mathematical argument about dynamics close to the boundary between stability and instability. Second, we used numerical methods applied to a model given by Law et al. (2012) over wide ranges of assumptions about predator–prey mass ratios, life histories, and the forms and intensity of fishing. This model is based on an extension of the McKendrick–von Foerster equation to include a diffusion term (Datta et al. 2010) and a model for size-dependent reproduction (Hartvig et al. 2011). Our results showed that the model of Law et al. (2012) robustly predicted that size spectra should conform to TL, density-mass allometry, and variance-mass allometry. Moreover, simulation of a size-at-entry fishery, in which only fish above a minimum body mass can legally be caught, disrupted these relationships more than did balanced harvesting.

## Methods

Size spectrum models are known to have equilibrium (steady state) solutions *u*_*s*_(*x*) and, in some cases, periodic solutions *u*(*x*, *t*) (Benoît and Rochet 2004; Andersen and Beyer 2006; Datta et al. 2011). The variable *u* represents the density (per unit volume of water) of organisms of body mass *w* = *w*_0_e^*x*^, where *w*_0_ is an arbitrary mass, say that of an egg. Throughout *u* is thought of as a function of the logarithm *x* of body mass *w*.

The solution of interest in this article was the periodic one, as this provided variation in density over time. We defined the mean *M*(*x*) and variance *V*(*x*) of the non-dimensionalized density at body size *x* over time as:


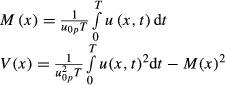


Here, *u*_0*p*_ was a reference density corresponding to the density of plankton at fish-egg size (Table 1). In the equilibrium case, *M*(*x*) equaled the equilibrium density *u*_*s*_(*x*) and *V*(*x*) was zero.

**Table 1:** Model parameters and values

Model parameter	Symbol	Value
Dynamic size spectrum		
Biomass conversion efficiency	*K*	0.2
Search rate scaling exponent	*α*	0.8
Search rate constant	*A*	600 m^3^ year^−1^g^−α^
Preferred log predator-to-prey mass ratio	*β*	5
Diet breadth	*σ*	1.5
Intrinsic mortality constant	*μ*_*i*,0_	1 year^−1^
Intrinsic mortality scaling exponent	*ξ_i_*	0.25
Mass at onset of senescent mortality	*w*_0_ exp(*x*_*s*_)	440 g
Senescent mortality scaling exponent	*ξ*_*s*_	5
Fish life history		
Mass of egg	*w*_0_	0.001 g
Mass at maturation midpoint	*w*_0_ exp(*x_m_*)	150 g
Asymptotic mass	*w*_0_ exp(*x*_∞_)	1000 g
Reproduction function exponent	*ρ*	0.2
Controls the width of the transition from immaturity to maturity	*ρ_m_*	10
Fixed plankton size spectrum		
Maximum body mass of plankton	*w*_0_ exp(*x_p_*)	0.001 g
Plankton density at *x*_0_	*u*_0*p*_	100 m^−3^
Plankton size spectrum exponent	−*γ*	−2
Fishing		
Fishing mortality	*F*	0–2.5 year^−1^
Minimum capture size	*w*_0_ exp(*x*_*f*_)	150 g

Where the model had periodic behavior, but was close to the bifurcation from a stable equilibrium, analytical methods were used to predict the exponent for TL in an idealized form of the model (Datta et al. 2011; Plank and Law 2011). To investigate what happened in the more realistic model of Law et al. (2012), and when the system was not close to the bifurcation point, numerical methods were used to solve the model until it converged either to a stable equilibrium or to a periodic solution. In the periodic case, convergence was tested by numerically integrating the system for a sufficient period of time and checking for periodic behavior of the total biomass of the system. The mean *M*(*x*) and variance *V*(*x*) of the periodic solution were then calculated using the above expressions. These expressions would be exact if the length of the integration *T* were an exact multiple of the period of the solution. However, the period was not known a priori and was difficult to determine accurately. Hence, in numerical calculations, an approximate estimate for the period was obtained and then *T* was set to be 10 times this estimate. The results were also checked for convergence with respect to *T*.

A linear relationship was fitted between the logarithm of the mean density ln *M*(*x*) and the logarithm of body size *x* using standard linear regression:


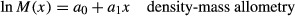
(1)

Here, *x* = 0 corresponds to *w*_0_ = 0.001 g (Table 1). Where the attractor was a periodic solution, relationships between the variance of density and body size, and between the variance and mean density were also fitted by the same method:


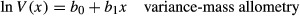
(2)


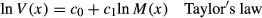
(3)

The regression parameters *a*_0_, *b*_0_, and *c*_0_ are referred to as intercepts. The parameters *a*_1_, *b*_1_, and *c*_1_ correspond to the exponents of density-mass allometry, variance-mass allometry, and TL, respectively. The body size variable *x* spanned the range 0 ≤ *x* < *x*_*s*_ (*x*_*s*_ = 13) in steps of size δ*x* = 0.1 giving *n* = 130 data points for each linear regression. The same range for *x* was used for each combination of parameter values investigated.

For the parameter values used by Law et al. (2012), the size spectrum converged to a stable equilibrium. Increasing the mean predator-to-prey mass ratio (PPMR) *β* or decreasing the diet breadth *σ* generally moved the model into regions of parameter space for which the equilibrium was unstable and the attractor was periodic (Plank and Law 2011). We adopted a smaller value for the diet breadth *σ* than that used by Law et al. (2012) to focus attention on periodic solutions. All other parameter values were the same as those used by Law et al. (2012) unless otherwise stated (see Table 1). The life history parameters in Table 1 correspond to a species that eats both plankton and smaller fish, has an egg mass of *w*_0_ = 1 mg, a maturity ogive with a midpoint at 150 g, and an asymptotic mass of 1000 g. The size spectrum parameters in Table 1 have the following meanings. Twenty percent of prey biomass is converted into predator biomass (*K* = 0.2). Predator search rate is proportional to *w*^α^, where the exponent *α* = 0.8 is widely used in size spectrum models (Benoît and Rochet 2004; Andersen and Beyer 2006; Blanchard et al. 2009) and is based on calculations of how cruising speed of fish scales with body mass (Ware 1978). Predators typically consume prey items whose body mass is between 1/10 and 1/1000 of their own mass (*β* = 5, *σ* = 1.5). The largest source of mortality is predation (*A* = 600 m^3^ year^−1^ g^−*α*^), but there is in addition an intrinsic mortality rate that is proportional to *w*^−0.25^ (Brown et al. 2004) and a senescent mortality rate that applies to fish larger than 440 g. The senescent mortality rate is needed in a single-species model to prevent the buildup of large organisms that have no predators, but may not be needed in a community model containing other species that grow to larger body masses and continue to act as predators (e.g. Andersen and Beyer 2006). See Law et al. (2012) Appendix for full details of the model equations and parameters.

## Results

In an idealized form of the model, designed to be mathematically tractable (Datta et al. 2011), the amplitude of perturbations to the equilibrium was a fixed proportion of the mean density, near the bifurcation between equilibrium and periodic behavior. If the ratio of perturbation amplitude to mean density (which is proportional to the coefficient of variation) were exactly constant, then the variance would be exactly proportional to the mean squared, which is TL with an exponent of exactly 2. The results of Plank and Law (2011) showed that the ratio of perturbation amplitude to mean density was not constant, but increased slowly with body size, and therefore decreased slowly with mean density. This predicted that the size spectrum should obey TL with an exponent of 1.87, slightly <2 (see Appendix 1A for details). To obtain this analytical result, it was necessary to assume an infinite range of body masses, and reproduction was not explicitly included. However, the result provided a formal basis for the numerical calculations that follow.

**Figure 1:**
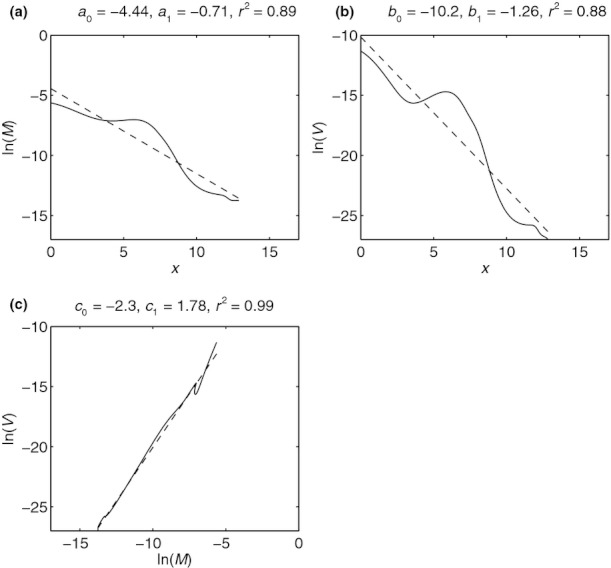
The model solution (solid curve) and the fitted linear regression (dashed straight line) for: (a) mean population density against log body size (*x*) (or density-mass allometry (1)); (b) variance of population density against log body size (or variance-mass allometry (2)); (c) variance of population density against mean population density (or Taylor's law (3)). Parameter values were as in Table 1.

In numerical solutions of the model of Law et al. (2012), the three fitted linear regressions (1), (2), and (3) for the parameter values given in Table 1 all had coefficient of determination *r*^2^ > 0.88, indicating that the model solutions fitted a linear regression reasonably well (Fig. 1). The value of the TL exponent *c*_1_ was 1.78, which is close to the theoretically predicted value of 1.87. If all three power laws held exactly, the parameters of density-mass allometry, variance-mass allometry, and TL would be related exactly by *b*_1_ = *a*_1_*c*_1_ and *b*_0_ = *c*_0_ + *c*_1_*a*_0_. The fitted values of *b*_0_ and *b*_1_ agreed with the values of *b*_0_ and *b*_1_ estimated using these relationships to the number of significant figures given in Fig. 1.

**Figure 2:**
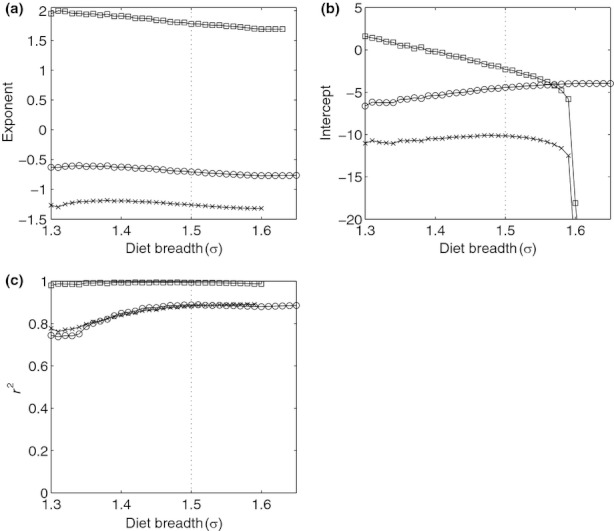
Effect of varying the diet breadth *σ* on the fitted linear regressions (circles – density-mass allometry (1); crosses – variance-mass allometry (2); squares – Taylor's law (3)): (a) exponents *a*_1_, *b*_1_, and *c*_1_; (b) intercepts *a*_0_, *b*_0_, and *c*_0_; (c) *r*^2^. All other parameter values were as in Table 1. Dashed vertical line shows the value of *σ* = 1.5 corresponding to the results shown in Figure 1.

To check how sensitive these results were to model parameters, we varied several key parameters within ranges that gave periodic behavior. For each set of parameter values, we calculated the regression parameters and the value of *r*^2^ for (1), (2), and (3). For example, when we varied the diet breadth *σ*, the fitted exponents were fairly insensitive to the value of *σ* (Fig. 2a). The intercept of density-mass allometry (1) was also fairly insensitive. However, the intercepts of variance-mass allometry (2) and TL (3) were more sensitive (Fig. 2b) because the model underwent a bifurcation at *σ* ≍ 1.59. When the parameter *σ* went above the bifurcation point, the system converged to a stable equilibrium rather than a periodic solution. The variance was therefore zero in this region of parameter space, and the curves (2) and (3) representing variance of population density were no longer defined, hence absent from the graph. As *σ* approached the bifurcation point from below, the intercepts of (2) and (3) tended to −∞. The values of *r*^2^ (Fig. 2c) showed that the model solutions fitted TL (3) very closely (*r*^2^ > 0.98) for all values of σ tested. Relationships (1) and (2) fitted less well, particularly for lower values of *σ* but still had *r*^2^ > 0.74 for all values tested. The fitted regression parameters agreed well with the parameters estimated via *b*_1_ = *a*_1_*c*_1_ and *b*_0_ = *c*_0_ + *c*_1_*a*_0_ across the range of values of *σ* shown in Fig. 2.

We repeated this analysis using several different model parameters: the preferred log PPMR (*β*); the maturation midpoint (*x*_mat_); the maximum size of the fixed plankton spectrum (*x*_*p*_); and the slope of the fixed plankton spectrum (*γ*). The maturation midpoint *x*_mat_ is the body mass at which 50% of fish have reached reproductive maturity. When we varied this parameter, we also varied the asymptotic (maximum) body mass (*x*_∞_) so that the ratio of asymptotic mass to mass at maturation was constant. Some theoretical and empirical evidence suggests that this ratio varies little across species (Beverton 1992; Charnov 1993). Varying each of these parameters changed the linear regression statistics (Table 2) remarkably little and the linear regressions fitted well (*r*^2^ > 0.7 for the relationships with body mass; *r*^2^ > 0.96 for TL) in all cases. For many of the parameters investigated, the system underwent a bifurcation from periodic to equilibrium behavior at some point in the parameter range, giving intercepts that tended to −∞ at the bifurcation point as observed above.

**Table 2:** The effect of varying model parameters on the fitted relationships among body size, mean population density, and variance in population density

Parameter and range	Mean: body size (1)	Variance: body size (2)	Variance: mean (3)
Diet breadth *σ* [1.3, 1.65]			
Exponent	[−0.7, −0.6]	[−1.4, −1.2]	[1.7, 2]
Intercept	[−7, −4]	[−∞,−10]	[−∞, 2]
*r*^2^	[0.73, 0.89]	[0.76, 0.91]	[0.98, 1.00]
Preferred predator-to-prey mass ratio *β* [4, 6]			
Exponent	[−0.8, −0.5]	[−1.4, −1.1]	[1.6, 2.1]
Intercept	[−7, −4]	[−∞,−9]	[−∞**,** 3]
*r*^2^	[0.79, 0.90]	[0.81, 0.89]	[0.97, 1.00]
Maturation midpoint *x*_*m*_ [10.5, 13]			
Exponent	[−0.8, −0.5]	[−1.4, −1.2]	[1.7, 1.9]
Intercept	[−5, −4]	[−∞,−10]	[−∞,−2]
*r*^2^	[0.85, 0.98]	[0.85, 0.92]	[0.98, 1.00]
Maximum plankton size *x*_*p*_ [0, 3]			
Exponent	[−0.8, −0.6]	[−1.3, −1.2]	[1.6, 1.8]
Intercept	[−5, −3]	[−∞, −9]	[−∞,−2]
*r*^2^	[0.88, 0.98]	[0.88, 0.97]	[0.96, 1.00]
Plankton spectrum exponent −*γ* [−2.2, −1.5]			
Exponent	[−0.8, −0.6]	[−1.4, −1.2]	[1.7, 1.8]
Intercept	[−6, −4]	[−14, −9]	[−4, −2]
*r*^2^	[0.86, 0.90]	[0.86, 0.88]	[0.99, 1.00]

Lower bounds of −∞ for intercepts indicate that the variance tended to zero within the investigated parameter range.

Two different size-based fishing protocols affected these relationships differently. In a standard size-at-entry fishery, all fish below a certain minimum catch size are protected from fishing, in principle. We incorporated this into the model by assuming a constant fishing mortality rate *F* for all fish above this body mass. In a model of balanced harvesting, fish of a given body mass are harvested in proportion to their natural productivity (Garcia et al. 2012). We adopted the same measure of productivity as Law et al. (2012): productivity *P*(*x*) was defined to be the product of the somatic growth rate and the density of individuals of a given size at the unexploited equilibrium. The fishing mortality rate at logarithmic body size *x* was then set as *μ*_*f*_(*x*) = *FP*(*x*)/*P*_0_, where *F* is a constant and *P*_0_ is the productivity at the smallest harvested body mass, which was set to be 1 g. For each fishing scenario considered, for a range of levels of fishing intensity *F*, we also calculated the total time-averaged yield. This was the sustainable yield in the sense that it was calculated from the long-term state of the system.

**Figure 3:**
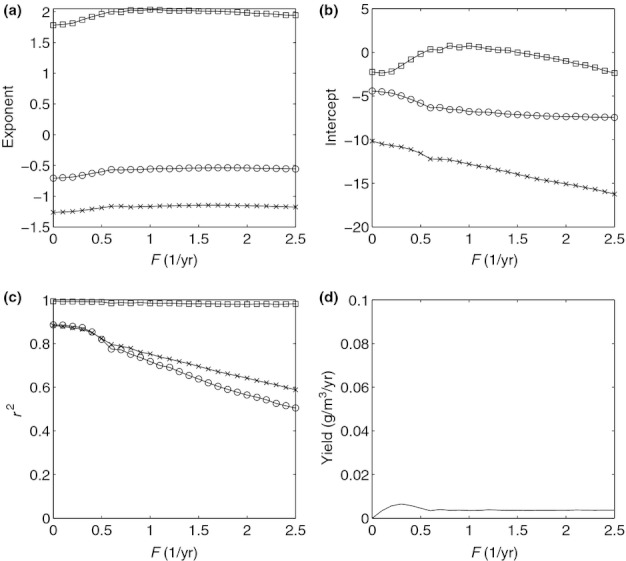
Effects of size-at-entry fishing on the fitted linear regressions (circles – density-mass allometry, (1); crosses – variance-mass allometry, (2); squares – Taylor's law, (3)): (a) exponents *a*_1_, *b*_1_, and *c*_1_; (b) intercepts *a*_0_, *b*_0_, and *c*_0_; (c) *r*^2^; (d) yield. Fishing mortality was zero below minimum catch size and was constant *F* above minimum catch size. In these graphs, minimum catch size was *x*_f_ = 11.9, which is the same as the mean maturation mass. Other parameter values were as in Table 1.

**Figure 4:**
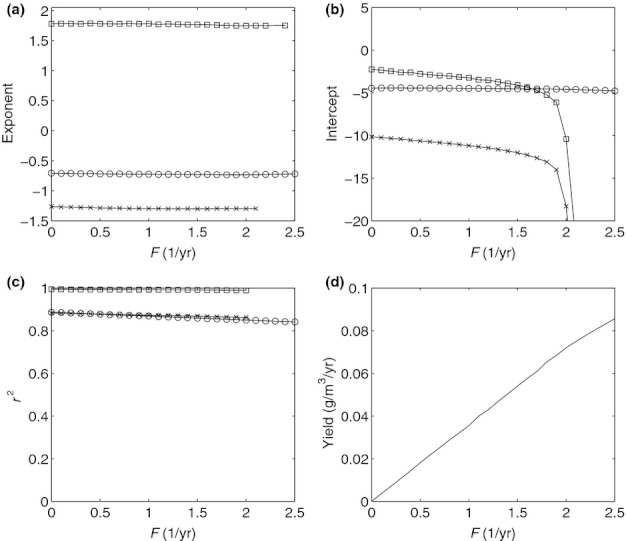
Effect of balanced harvesting on the fitted linear regressions (circles – density-mass allometry, (1); crosses – variance-mass allometry, (2); squares – Taylor's law, (3)): (a) exponents *a*_1_, *b*_1_, and *c*_1_; (b) intercepts *a*_0_, *b*_0_, and *c*_0_; (c) *r*^2^; (d) yield. Fishing mortality was proportional to natural productivity with constant of proportionality *F*. All parameter values were as in Table 1.

Under size-at-entry fishing, the system behaved periodically across the range of fishing intensity *F* we investigated (Fig. 3). Under balanced harvesting (Fig. 4), the system bifurcated to stable equilibrium behavior as the fishing effort increased (variance-mass allometry intercept *b*_0_ and TL intercept *c*_0_ tended to −∞ in Fig. 4b). Neither fishing method had a large effect on the exponents of the allometric relationships investigated. Balanced harvesting did not have a substantial effect on *r*^2^ (Fig. 4c), but size-at-entry fishing substantially reduced *r*^2^ for density-mass allometry and variance-mass allometry. This fishing protocol substantially altered the size spectrum away from the density-mass allometric power-law (1). The size-at-entry fishery had a greater effect on the intercept *a*_0_ of equation (1) (compare circles in Fig. 3b and Fig. 4b). A lower intercept and a comparable exponent indicated that size-at-entry fishing reduced the biomass of the remaining stock to a greater extent than did balanced harvesting. Finally, the yield for size-at-entry fishing (Fig. 3d) with these parameter values did not exceed around 0.065 g m^−3^ year^−1^. In contrast, balanced harvesting gave yields of up to 0.85 g m^−3^ year^−1^ (Fig. 4d). We have not optimized either fishing protocol to find the level of fishing effort or (in the case of size-at-entry) the minimum catch size that would give the maximum sustainable yield. Nevertheless, these results supported the findings of Law et al. (2012), which showed that balanced harvesting gave a higher maximum sustainable yield than size-at-entry fishing, and disrupted the ecosystem's natural size structure much less.

## Discussion

Equilibrium properties of size spectrum models have been the subject of much recent research (Andersen and Beyer 2006; Andersen et al. 2008; Blanchard et al. 2009; Datta et al. 2010, 2011; Plank and Law 2011). The behavior of size spectra away from equilibrium has, in comparison, received less attention, although real systems often do not operate at equilibrium. Zhang et al. (2012) showed that incorporating species diversity (via the asymptotic body size trait) into the community model promoted stability and reduced the amplitude of oscillating solutions. Rochet and Benoît (2012) showed that size-selective fishing increases the amplitude of oscillations in the size spectrum.

Here, in one of the first studies to quantify the behavior of a size spectrum model away from equilibrium, we investigated allometric (power-law) relationships among body mass, mean population density, and variance of population density in a model of marine size spectra. We used mathematical arguments to predict that populations should conform to these allometric relationships in an idealized form of a size spectrum model. Within ecologically realistic ranges for model parameters, numerical calculations confirmed that these relationships were remarkably robust, especially the relationship between variance and mean of population density (TL). Even when the allometric relationships of the mean and variance of population density to body size were slightly weaker, TL still appeared to hold. The coefficient of determination (*r*^2^) for TL was >0.96 in all cases studied and the exponent was always between 1.6 and 2.1 and usually slightly <2.

Some size spectrum models do not explicitly include reproduction, but assume a constant density of individuals with body mass corresponding to the mass of an egg (Benoît and Rochet 2004; Blanchard et al. 2009; Law et al. 2009; Zhang et al. 2012). Other models explicitly couple the density of eggs to reproduction by mature individuals (Andersen et al. 2008; Hartvig et al. 2011; Rochet and Benoît 2012). Analytical results from a model without reproduction (Plank and Law 2011) predicted that the size spectrum should obey TL approximately, with an exponent slightly <2. However, the goodness-of-fit and the exponents of TL and variance-mass allometry could be assessed more accurately across a range of parameter values by avoiding the artificial constraint of zero variance in the density of eggs. In our numerical calculations, we therefore used a model that explicitly included reproduction (Law et al. 2012). Under this model, there is a natural interplay between variance in the density of eggs and the variance in the density of mature adults.

Normally, TL applies to a set of populations, each with its own mean and variance of density. TL and variance-mass allometry have not before been demonstrated within a model of a single population. For instance, earlier work has shown that TL with an exponent 2 emerges in the discrete logistic growth model, *N*_*t*+1_ = *rN*_*t*_(1 − *N*_*t*_/*K*), when different populations have different carrying capacities *K*. If *N*(*t*) is a solution to the model with carrying capacity *K* then *aN*(*t*) is a solution to the model with carrying capacity *aK*. A straightforward consequence is that the variance must be proportional to the mean squared across a set of populations with different values of *K* (Ballantyne 2005). Size spectrum models are different in that they disaggregate organisms by body mass allowing individuals to grow, often over several orders of magnitude of body mass, over the course of their lives as a result of eating smaller individuals. Hence, all the data on mean and variance of abundance come from organisms of different body sizes within one population or community. The models we used did not assume a particular carrying capacity. The long-term biomass dynamics instead arose as a consequence of the interaction between the dynamic size spectrum and its (assumed fixed) resource spectrum, as well as between different body sizes (predator and prey) within the dynamic spectrum. The finding that these dynamics conform to TL so closely across a wide range of parameter values was therefore new and surprising.

Balanced harvesting, that is, spreading fishing effort across as wide a range of species and body sizes as possible, has recently been proposed to be a more efficient and less harmful way of fishing than traditional fishing regulations such as size-at-entry (Zhou et al. 2010; Garcia et al. 2012). In the context of a single-species size spectrum model, one model for balanced harvesting has been to match fishing effort with natural productivity across the range of body sizes in the population (Law et al. 2012). In practice, this means focusing more effort on the more productive, smaller fish and reducing fishing intensity on the larger fish. A key potential advantage of this balanced harvesting regime is that it is predicted to alter the relationship between mean population density and body mass much less than more selective fishing methods (Law et al. 2012). Our results added further weight to this conclusion by showing that balanced harvesting also disrupted the variance-mass allometry less than size-at-entry fishing.

## Appendix A. Theoretical basis for Taylor's law in the size spectrum model

The periodic behavior of the model arises via a Hopf bifurcation from stable equilibrium to stable periodic orbit. At the bifurcation, a complex conjugate pair of eigenvalues *λ* has zero real part *λ* = ±*iω*. Close to the bifurcation, the limit cycle solution can be expressed as a small perturbation to the steady state *u*_*s*_(*x*):

(A1)

where *ε*(*x*) is the eigenfunction associated with the eigenvalue *λ* = *iω* and the overbar denotes complex conjugate. *B* is a complex constant whose magnitude grows in proportion to, where *μ* is the bifurcation parameter and *μ** is its bifurcation point (Strogatz 1994).

From equation (5), the mean population density is simply *M*(*x*) = *u*_*s*_(*x*) and, using *T* to denote the period of the limit cycle, the variance is

(A2)

Since

for any non-zero integer value of *n*. Analytical expressions for the eigenfunction *ε*(*x*) are available only in certain idealized forms of the model. In the special case where there is no reproduction, body sizes span an infinite range, and a constraint *α* = *γ −* 1 is placed on the model parameters (*α* is the power-law exponent for the scaling of predator search rate with body size and *γ* is the slope of the steady-state size spectrum), the eigenfunctions are plane waves of fixed amplitude (Datta et al. 2011):

where *k* is a real-valued constant. In this case, the magnitude of the eigenfunction is constant |*ε*(*x*)| = 1, which implies by equation (A2) that Taylor's law (TL) holds exactly with an exponent of 2.

When the model parameters do not satisfy the constraint *α* = *γ* − 1, numerical results (Plank and Law, 2011) showed that, for parameter values comparable to those in Table 1, the magnitude of the eigenfunction |*ε*(*x*)| increases with *x*, approximately proportionally to e^*ax*^ where *a* = 0.07. The mean density *M*(*x*) is proportional to e^(1 − *γ*)*x*^ where *γ* = 2.05, so

Hence, the results of Plank and Law (2011) combined with equation (A2) predict that the size spectrum should obey TL:

(A3)

for some constant *C*. The predicted exponent for TL is 2 + 2*a*/(1 − *γ*) = 1.87. The fitted exponent in the model of Law et al. (2012), which includes reproduction, (Fig. 1c) is 1.78. From equation (A3), the intercept *c*_0_ for TL (3) is

Close to the bifurcation, the magnitude of *B* scales with  (Strogatz 1994). Hence, the intercept for TL is

This explains the logarithmically shaped decay to −∞ of the intercept *c*_0_ as the parameter approaches the bifurcation point in Fig. 2b and 4b.

These arguments were based on an idealized form of the model that assumed an infinite range of body sizes and did not explicitly include reproduction. Nevertheless, they gave some analytical insight into why TL applied in a model of size spectra, and they provided qualitatively accurate predictions of the exponent and intercept.
